# Harnessing Mechanosensation in Next Generation Cardiovascular Tissue Engineering

**DOI:** 10.3390/biom10101419

**Published:** 2020-10-07

**Authors:** Gloria Garoffolo, Silvia Ferrari, Stefano Rizzi, Marianna Barbuto, Giacomo Bernava, Maurizio Pesce

**Affiliations:** 1 Unità di Ingegneria Tissutale Cardiovascolare, Centro Cardiologico Monzino, IRCCS, 20138 Milan, Italy; silvia.ferrari@ccfm.it (S.F.); Stefano.rizzi@ccfm.it (S.R.); marianna.barbuto@ccfm.it (M.B.); giacomo.bernava@ccfm.it (G.B.); maurizio.pesce@ccfm.it (M.P.); 2 PhD Program in Translational and Molecular Medicine—DIMET, Università di Milano-Bicocca, 20126 Milan, Italy; 3 PhD Program in Translational Medicine, Università degli studi di Pavia, 27100 Pavia, Italy

**Keywords:** mechanosensing, cardiac regeneration, tissue engineering

## Abstract

The ability of the cells to sense mechanical cues is an integral component of ”social” cell behavior inside tissues with a complex architecture. Through ”mechanosensation” cells are in fact able to decrypt motion, geometries and physical information of surrounding cells and extracellular matrices by activating intracellular pathways converging onto gene expression circuitries controlling cell and tissue homeostasis. Additionally, only recently cell mechanosensation has been integrated systematically as a crucial element in tissue pathophysiology. In the present review, we highlight some of the current efforts to assess the relevance of mechanical sensing into pathology modeling and manufacturing criteria for a next generation of cardiovascular tissue implants.

## 1. Introduction

Pathologic evolution of the cardiovascular system is not only accompanied by modifications in cellular metabolism, aging, epigenetics and risk condition-associated defects but also by fundamental changes in cells/tissues architecture, motion and geometry. As an example, the chronic setting of ischemic disease in the heart results in variations in the physical–chemical characteristics of the extracellular matrix, which lead to changes in the structure of the myocytes contractile apparatus (the sarcomere) causing, alternatively, systolic or diastolic heart failure [1]. Another example is represented by the chronic alterations of the extracellular matrix in the cardiac valves, where the inflammatory condition consequent to lipid accumulation primes the interstitial cells to acquire a matrix pro-remodeling phenotype, causing tissue calcification [2] and changes in mechanical compliance [3].

Apart from neo-vascularization observed in consequence of local activation or repair mechanisms (e.g., wound healing, ischemia-dependent angiogenesis), the cardiovascular system is not endowed with extensive regeneration ability. A typical example is the adult myocardial tissue, where the presence of cardiogenic cells able to replenish the contractile cells after injury has been dismissed after tense discussions lasting for about two decades [4]. This has created a lot of disappointment concerning the feasibility of regenerating the heart to prevent the consequences of heart failure.

After the initial enunciation of the tissue engineering principles by Langer and Vacanti in 1993 [5], research has shown the limitations of a simplistic approach to generate tissues or entire organs by combining cells and materials without controlling the cellular microenvironment not only from a compositional/chemical point of view but also mechanically. In fact, the demonstration that cells are able to decrypt mechanical properties of the surrounding environment and translate them into biophysically regulated gene expression patterns [6] determines a new level of complexity in which cells interact with scaffolds and materials employed to generate tissues. In the present article, we discuss aspects of the mechanical-dependent pathological programming of cardiovascular cells to come up with a revision of the current strategies to contextualize physiologic tissue mechanics in cardiovascular tissue engineering procedures.

## 2. Myocardium

### 2.1. Matrix Mechanics as a Regulatory Factor for Cardiac Fibrosis

Cardiac fibrosis, characterized by accumulation of extracellular matrix (ECM) production and degradation, results in a progressive evolution of myocardium into a non-contractile fibrotic tissue. Indeed, this pathological remodeling leads to a replacement of myocytes with a stiff fibrotic tissue, affecting the myocardial compliance and accelerating the progression to heart failure. This process is mediated by cardiac fibroblasts (CFs), which physiologically are quiescent cells responsible for ECM homeostasis [7]. Following acute myocardial injury, CFs respond by transforming into activated myofibroblasts (MFs); they express elevated levels of pro-inflammatory/fibrotic factors, which directly contribute to inflammatory cell infiltration and fibroblast proliferation [8]. Therefore, MFs secrete high levels of matrix metalloproteinase and other ECM degrading enzymes that promote fibroblast migration to the site of injury, and they contribute to the production and deposition of collagens. All these reparative mechanisms are necessary to maintain the structural integrity of the heart and prevent myocardial rupture [9]. These processes, initially adaptive, lead to a complete pathological change in the myocardial architecture, with significant consequences on cardiac functions. For example, fibrotic ECM increases ventricular stiffness and, therefore, contractile dysfunction of cardiac myocytes [10]. Changes in the mechanical proprieties of the heart are in turn responsible for CF activation, and can directly affect fibroblasts or induce paracrine signals from mechanically stressed cardiomyocytes [11]. Various in vitro evidence shows that mechanical stimuli, derived from tissue stiffness or elevated stretch, are translated into biochemical signals, which regulate the transcriptional activity of genes involved in myofibroblast activation [12,13,14,15]. The relevance of mechanical cues in the pro-pathological setting of CFs emerges also from experiments demonstrating that an environment with altered mechanical compliance irreversibly affects the long-term cell memory prompting cells to express disease-related genes even after reversion of matrix mechanics to physiological conditions [16,17]. Hence, this raises the possibility that mechanical cues control CF phenotype by regulating epigenetic landscape and thus promoting a chronically activated phenotype that contributes to cardiac fibrosis [18] through recruitment of innate immunity cells [19]. Recently, a functional interaction between fibroblasts and macrophages to coordinate tissue repair after injury has been demonstrated. Local remodeling of collagen fibers by fibroblasts provides physical cues to attract macrophages at long distances [20]. This suggests that mechano-dependent epigenetic alterations may be responsible for the persistent activated phenotype of MFs, for the consequent increase in matrix elasticity of the myocardium and, therefore, also for the onset of a potent cardiac inflammatory response. Evidence of the existence of a mechanical control of cardiac fibroblasts behavior can open new paths in the development of more efficient therapeutic strategies restoring the normal mechanosensing of CFs, also taking in consideration inflammatory and metabolic cues.

### 2.2. In Situ Heart Regeneration: New Approaches for Limiting Progression of Cardiac Remodeling and Failure

To date, the only possible therapy for patients with end-stage heart failure consists of implantation of a left ventricular assist device (LVAD) or transplantation of an entire donor heart. However, none of these solutions allows recovering structural and/or physiological integrity of the cardiac muscle—for example by restoring coordinated contraction and coherent electrical coupling. 

Lower vertebrates such as zebrafish demonstrate an impressive natural capacity for cardiac regeneration throughout life. This has been also well documented in other various non-mammalian vertebrate’s species [21,22]. By contrast, in mammalians, there is an intrinsically limited ability of cardiomyocytes (CMs) to self-renew [23,24] with a rate capacity of ~1% per year [25]. Therefore, a mature heart has a low ability to compensate for the pathological loss of cardiac myocytes in consequences of acute injuries such as a myocardial infarction (MI), or chronic ischemia setting such as coronary artery disease.

Direct cardiac reprogramming has emerged as a promising therapeutic approach for regenerating the damaged heart by directly converting endogenous cardiac fibroblasts into cardiomyocyte-like cells using viral vectors overexpressing cardiac master genes [26,27] or micro-RNAs (miRNAs) [28]. CFs constitute approximately 10% of all cardiac cells in the heart [29], thus representing a potential source of cardiomyocytes for cardiac regeneration [30,31,32]. This conversion may reduce fibrosis and scar formation by decreasing the pool of activated fibroblasts after MI, thus preventing the adverse matrix remodeling that contributes to contractile dysfunction and arrhythmogenesis [33]. Different gene editing tools, including zinc fingers nuclease (ZFNs) and clustered regularly interspaced short palindromic repeats (CRISPR) systems, were developed to study cardiovascular diseases. Several studies have shown that cardiomyocytes can be edited in the post-natal murine heart using the viral delivery of CRISPR/Cas9 components generating transgenic animal models [34]. For example, CRISPR/Cas9 was used to reprogram human fibroblasts into induced-cardiac progenitors, which can differentiate into different cell types in vitro, including cardiomyocytes [35]. The efficiency of gene editing depends on the employed delivery strategy, which can be used ex vivo, using a “patient-to-patient” approach by editing autologous cells and transplanting them back in the patients or in vivo by direct injection of CRISPR/Cas9 in the damaged tissues. Despite its simplicity, scalability and affordability, there are biological, technical and ethical issues that limit the therapeutic application of this tool. 

An alternative to the reprogramming approach is the delivery of drugs promoting re-activation of the CM cell cycle [36]. In recent years, investigators have identified signaling pathways that may be exploited to induce cardiomyocytes cell cycle reactivation promoting cardiac tissue regeneration. A pathway that connects directly proliferation of myocytes and mechanosensation is the Hippo signaling pathway. This pathway, named after the gigantism of the organ size of the Hippopotamus, is a known controller of the organ size in several vertebrates and invertebrates, depending on the balance between transcriptionally active components (YAP/TAZ transcription factors), directly connected to cell proliferation, and their inactivation by phosphorylation cascades [37]. In the mammalian heart, the pathway may have an ambivalent role in controlling cardiac development/patterning and damage associated fibrosis. Indeed, YAP nuclear localization is necessary to maintain an elevated level of immature myocyte proliferation until shortly after birth, when activation of the Hippo kinase cascade keeps the transcription factor outside the nucleus restraining myocytes proliferation [38,39]. On the other hand, hyperactivation of the YAP-dependent gene signature has been observed in the transition of cardiac fibroblasts toward myofibroblasts, thus connecting activation of the transcriptional Hippo pathway to cardiac fibrosis [40].

How could manipulation of Hippo signaling be exploited to regenerate the heart? YAP in adult myocytes is sequestered out of the nucleus, not only by the activity of the Hippo kinase pathway, but also by association of the protein to components of the cytoskeleton involved in extracellular stiffness sensing (e.g., dystroglycan) [41,42]. Given that the dystroglycan complex is connected to ECM receptors and the contractile apparatus, it is tempting to speculate that altering the biophysical characteristics of the extracellular matrix could lead to the release of myocytes from the mitotic block by reverse sequestering of YAP by cytoskeleton. In keeping with this hypothesis is the evidence that chemically modifying the elastic modulus of the ECM in juvenile mice, the proliferative phase of myocytes in the heart is significantly prolonged [43]. Pharmacological inhibition of the collagen and elastin cross-linking enzymes, inducing cardiac ECM stiffness reduction, largely prevented the fibrotic response to injury and improved CM regeneration capacity [43]. Therefore, direct activation of YAP in CMs, using pharmacological drugs/miRNAs specifically targeting the Hippo signaling [44,45], could represent a useful strategy to promote cardiac repair and regeneration in situ. 

Another way to harness the Hippo pathway to treat cardiac diseases (e.g., cardiac fibrosis and heart failure) depends on the function of the YAP/TAZ transcriptional complex in cardiac fibroblasts [46]. In this particular setting, preventing transcriptional activity of these factors by selective drugs may help to decrease the proliferation of these cells and their commitment into inflammatory/matrix remodeling phenotypes, thus limiting progression of maladaptive myocardial remodeling. Evidence in vivo demonstrated that YAP inhibition improved cardiac function by targeting myofibroblast transdifferentiation and attenuating matrix remodeling [47]. 

Several miRNAs are known for their cardiac anti-fibrotic activities and are dysregulated in myocardial injury models (Table 1). Among these, miR-29, often defined as a “cardioprotector”, is able to downregulate profibrotic proteins, such as collagens. This miR-29 has found to be downregulated in infarcted hearts [48]. Bioinformatics approaches led by Duisters et colleagues [49] identified two miRNAs, miR-133 and miR-30, as crucial players in matrix remodeling since they negatively regulate CTGF expression. This latter, indeed, promotes ECM synthesis with subsequent fibrosis progression and, intriguingly, is a target of the YAP/TAZ complex. This is an interesting example of how the development of fibrosis in the heart might be under mechanosensing-dependent control.

Not only YAP, but also other mechanical sensors seem to be implicated in regulating cardiac function and pathological progression of cardiac diseases. For example, integrins and the angiotensin II type I (AT1) receptors are involved in mechano-dependent cellular responses, such as differentiation, ECM remodeling, inflammation [52,53] and their pharmacological inhibitors emerge as potential therapeutic effectors to prevent heart damage [54]. 

Both viral- and non-viral-based vectors have been used to delivery genetic materials to the heart. However, gene therapy has shown various limitations and severe side effects. As an example, none of the tested gene delivery systems is 100% cardiac specific and can activate the innate immune system with significant dose-related toxicity [55]. Additionally, nonviral gene transfer approaches, including circular plasmid DNA vectors or synthetic modified mRNA, showed disadvantages due to their low gene delivery efficiency [56]. Moreover, implantation of cells with cardiomyogenic and/or angiogenic characteristics resulted to be unsuccessful due to the reduced intramyocardial cellular retention and limited cardiac function improvements [57].

Tissue engineered scaffolds and hydrogels might represent an alternative to drug delivery vehicles to treat cardiac disease. Engineered cardiac patches can be used as off-the-shelf products to mimic the native ECM; they further provide mechanical support and can release drugs into the region of infarction. Acellular injectable scaffolds show different advantages, such as a limited immune reaction, low cost and extended shelf life. Because of their tissue-like biological, chemical and mechanical properties, hydrogels represent potential carriers to release drugs/miRNAs into cardiac tissue. Hydrogels are 3D hydrophilic polymer networks with tunable, compliant and biomimetic behavior well suited for drug delivery [58]. “Intelligent hydrogels” respond to more than one environmental stimulus, such as pH, temperature, enzyme activity, electric or magnetic field or tissue composition, with changes in physical and chemical properties [59]. Moreover, these new materials with low substance viscosity and slow gelling property enable catheter delivery without an invasive surgery intervention. Thanks to their versatility, responsive hydrogels could be used as a cell-free scaffold in order to preserve tissue, promote self-regeneration and reduce negative remodeling after MI. An example is the injection of acellular alginate-based hydrogels in the myocardial tissue that prevented left ventricular (LV) remodeling acting as an artificial extracellular matrix in a swine model of MI [60]. A pilot study of 27 patients treated with the same temporary bioabsorbable cardiac scaffold showed positive outcomes in terms of tolerability, without device-related adverse events [61]. When injected, this hydrogel replaced the damaged extracellular matrix reducing LV dilation and infarct expansion, probably by decreasing the matrix stiffness of MI tissue. Injectable hydrogels can act directly affecting ECM compliance of the tissue or used as a scaffold for the delivery of short molecules/drugs. For example, multi-stimuli responsive hydrogels have the capacity to achieve site-specific chemotherapeutics delivery for improving drug efficacy and limiting toxic side effects [58]. An example is represented by hydrogels based on the hydrophobic amino acid residues L-phenylalanine and L-valine incorporated with magnetic nanoparticles [62].

**Figure 1:**
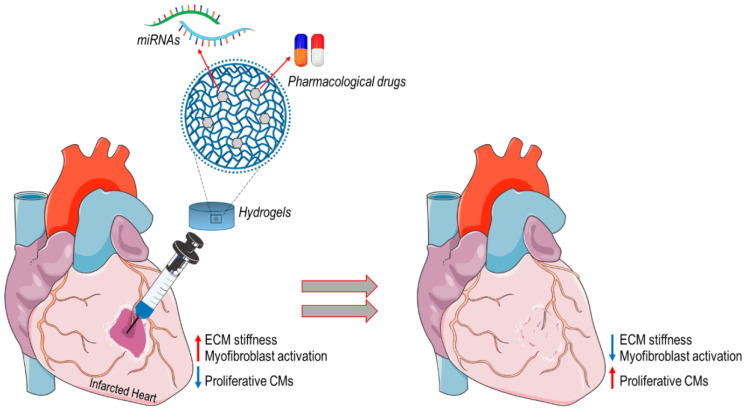
An example of “intelligent hydrogel” application for treating myocardial infarction. Hydrogels with defined mechanical compliance could be used as cell-free scaffolds, functionalized with pharmacological drugs or miRNAs decreasing ECM synthesis and cardiac myofibroblast activation (e.g., miR-29, miR-133 or drugs interfering with YAP transcriptional activity) or inducing CM reactivation of cell cycle to promote tissue regeneration (e.g., collagen/elastin cross-linking inhibitors, YAP/HIPPO signaling inhibitors).

Based on this evidence, we hypothesize that the use of “intelligent hydrogels”, supporting mechanical myocardial and releasing drugs/miRNA able to inhibit cardiac fibroblast activation and/or induce reprogramming into CMs could represent the awaited solution to prevent post-infarct myocardial remodeling and promote myocardial regeneration in patients with cardiac ischemia (Figure 1).

## 3. Valves

### 3.1. The Synergy between Mechanotransduction and Epigenetic Regulation in Calcific Aortic Valve Disease

The aortic valve is composed of three leaflets attached to a fibrous ring integrated in the aorta; each of these leaflets is composed of a thick extracellular matrix, organized into three layers named, respectively, lamina fibrosa, lamina spongiosa and lamina ventricularis. All three layers have peculiar matrix compositions, which fulfill different mechanical functions. The lamina ventricularis is richer in elastin and is important for the recoil of the tissue during the transition from the open (systolic) to the closed (diastolic) conformation. The lamina spongiosa contains principally proteoglycans, and it is the softest and most amorphous layer with numerous cells, the valve interstitial cells (VICs) [63]. Finally, the lamina fibrosa consists of a dense network of collagen bundles arranged in an anisotropic fashion due to crossing and overlapping at specific valve portions such as the ”belly” and the edges that are the areas exposed to the maximal diastolic stress [64,65].

Although VICs are embedded in the most protective layer in the leaflet, they are continuously exposed to mechanical stress by wear and tear. In their normal function, these cells are engaged in renewing the extracellular matrix subject to continuous mechanical stress. However, in consequence of inflammation due e.g., to lipid accumulation, they can modify their phenotype into that of pro-inflammatory cells, potentially affecting the matrix structure and biophysical characteristics. As a matter of fact, the pathological evolution of the aortic valve—calcific aortic valve disease (CAVD)—is characterized by extensive deposition of hydroxyapatite nodules in the leaflet layers [2], which affects the physiological opening/closing cycles of the valve and, as a result, compromises the valve functionality with significant effects on the blood flow velocity and regurgitation. Although lipid accumulation, inflammation, risks and genetic factors are commonly considered the main effectors of the pathology [66], growing evidence shows that mechano-perception may play a crucial role in VIC activation into myofibroblasts and in the subsequent osteoblastic differentiation [67]. Wang and colleagues demonstrated that VICs act as tissue “mechanosensors” susceptible to mechanical cues. Indeed, transition of these cells from a quiescent to an activated status is reversible and dependent on the stiffness of the surrounding matrix [68]. These data suggest that VICs may be able to respond to changes in ECM mechanical properties in order to maintain the physiological level of matrix components. Further evidence of VIC phenotypic plasticity and mechanical adaptability comes from a study in which the elastic modulus of VICs cultured on soft and stiff gels was measured. In particular, the VIC elastic modulus was higher on stiff substrates, demonstrating that these cells react actively and dynamically to variations of compliance of the surrounding matrix [69]. Although little is known regarding the specific pathways involved in VIC mechanosensation, in a recent publication by our group, we showed the stiffness dependency of YAP nuclear translocation in pathologic VICs adhering onto polyacrylamide gels (PAGs) with controlled stiffness [3]. Interestingly, while VICs from calcific valves exhibited a sharp increase in YAP nuclear localization in response to slight elevations in the substrate elastic modulus, VICs obtained from valves with a fibrotic disease (valve insufficiency) were less responsive. This difference was interpreted as a readout of pathology-specific differences in cytoskeleton traction forces that cells exert when in contact with materials with specific mechanical features. In support of these findings, the highest amount of VICs with nuclear localized YAP was found in areas of the human calcific leaflets exhibiting the maximal stiffness, as verified by direct determination of the leaflet elastic modulus in transversal sections by atomic force microscopy [3]. 

CAVD is regulated also by hemodynamic cues [70], in particular by pulsatile shear stress, a force acting on leaflets endothelium in the direction of blood flow and essential for valvular homeostasis. Unidirectional laminar shear stress is directed against the ventricularis layer, while the fibrosa experiences disturbed flow and oscillatory shear stress, which is known to be involved in the onset of the pathology [71]. With the progression of the stenosis, due to the stiffening of the leaflets, wall shear stress across the aortic valve dramatically increases [72]. Shear stress is tightly associated with mechanotransduction inducing a cytoskeletal rearrangement in endothelial cells with alignment in the direction of flow with morphological and functional changes [73]. Ahamed and colleagues demonstrated that disturbed flow and oscillatory shear stress are able to activate latent TGF-β and bone morphogenetic protein (BMP) [74]. These mechanosensitive molecules, once bound to their receptors, promote Smad transcription factor nuclear translocation with consequences for osteogenic genes transcription [74].

Epigenetics refers to several mitotically and meiotically transmissible DNA/chromatin modifications, which do not affect the primary DNA sequence, but its function. Epigenetics controls genome functions through modifications of the chromatin structure, including interactions with miRNAs and long-coding RNAs (lnc-RNAs) [18]. Growing evidence, indeed, define miRNAs as new regulators of gene expression with different translational readouts in the development of cardiovascular diseases, including CAVD [75] (Table 2). Zhang and colleagues found that miR-30b in human calcific valves interfere with VICs osteoblastic differentiation by targeting Runx2, Smad1 and caspase-3 [76]. Moreover, VICs grown in calcific medium and treated with miR-30b precursor, exhibited lower levels of alkaline phosphatase (ALP) activity, Runx2 and osteocalcin expression. Furthermore, transfection of porcine activated VICs with miR-141 resulted in the recovery of physiological signaling of other regulators of valve calcification, bone morphogenic protein-2 (BMP-2) and ALP [77]. Since VICs respond to mechanical signals of the surrounding ECM, miRNAs interfering with calcific crystal deposition might represent potent therapeutic tools [78]. Another mechanism by which epigenetics exerts its control is DNA methylation, usually associated with gene expression silencing [18]. In a mouse model, Barrick and collaborators demonstrated that a decrease in epidermal growth factor receptor (EGFR) expression levels corresponded to a dysregulation of its 5-hydroxymethylation pattern leading to calcification. EGFR, indeed, physiologically inhibits BMP activation, and interfering with EFGR activity may promote VICs osteoblastic differentiation [79]. Moreover, studies carried out in human osteoblastic cells demonstrated that ALP activity is regulated by DNA methylation: ALP promoter methylation resulted inversely associated with the transcriptional levels of ALP positive cells [80].

Taken together, this evidence holds the promise that a pharmacological therapy for CAVD progression could be endeavored by treating with drugs impacting on the progression of the disease by targeting the ability of VICs to decrypt mechanical cues and, at the same time, maintaining a permissive epigenetic landscape for physiological activity of the valve cells. Compared to other cardiovascular disorders, there are still extremely limited options for drug-based therapeutic approaches to limit progression of valve diseases, most of which—e.g., statins [83,84,85,86,87]—are repurposed drugs with a generic beneficial effect on cardiovascular pathology more than on a specific valve pathophysiologic process.

### 3.2. How to Engineer New Valve Substitutes—New Approaches for an Unresolved Problem

The field of cardiac valve tissue engineering dates about 30 years back with the demonstration of the growth of endothelial cells onto the surface of glutaraldehyde-fixed animal valves (reviewed in [88]). The interest in this subject emerged to overcome the limitations of the existing replacements of the valves, consisting of mechanical and biological prostheses for a population with a continuously growing life-expectation. The preferred characteristics of an ideal valve replacement are (i) the ability to integrate in the host with a self-renewing capacity; (ii) to not to elicit immune responses by the host, thus eliminating the risk of tissue calcification and failure; (iii) to be able to grow in concert with the growing heart (particularly relevant in case of pediatric subjects).

Tissue engineered heart valves (TEHVs) have been proposed historically as a solution for some of these requirements, such as the ability to self-renew and be manufactured with cells of the patients, thus eliminating rejection or long-term immune responses [89,90]. Moreover, the possibility to embed cells into scaffolds, which might be gradually reabsorbed, provided a great hope that, once adapted to the new environment, cells would behave similarly to the native cells by optimally and stably integrating in the host environment and maintaining tissue geometries and functions even after reabsorption of the scaffolds. Unfortunately, this has not been the case, even using the most accurate and comprehensive designs and manufacturing abilities, thus limiting feasibility to large animal models and preventing effective human translation [91].

Problems inherent to the loss of tissue structure and mechanical coherence are the major limitations of TEHVs in the long term. These modifications are due to the biological activity of the cells embedded in the initial constructs, and those from the host that gradually populate the constructs over time after implantation. ”Compaction” and ”retraction” are the two most recurrently observed modifications of TEHV leaflets. The first effect is determined by the uncontrollable matrix remodeling activity of the cells that in the long term determine shrinking and thickening of the leaflets. The second effect is relative to loss of mechanical coherence of the leaflets that cause valve regurgitation. From a biological point of view, transformation of the cells of the valve tissue constructs into active myofibroblasts is one of the most important causes of both effects. Myofibroblasts, in fact, have potent matrix remodeling activity and exert strong pulling forces dependent on stress fibers [92] potentially affecting the geometry of the leaflets [93]. Research is now concentrated onto three different approaches to overcome this problem: in situ, in vitro and in vivo tissue engineering techniques [94].

In the in situ approach scenario, cell-free prostheses, ideally manufactured with biologically compatible polymers, are provided as ”off-the-shelf” implants ready to be used without pre-cellularization [95] and are tailored to be colonized by cells recruited from the bloodstream (e.g., granulocytes, monocytes, macrophages) contributing to tissue regeneration with an inflammatory-like wound healing process [96]. In this approach, the design of the scaffolds as controlled environments favoring a correct integration of cells, and instructing valve tissue generation through a developmental biology-like process, are extremely relevant [97]. Different biomaterials and scaffolds are designed with specific characteristics in order to induce valve tissue regeneration through direct interaction with proteins and cells. This new class of biomaterials, designed with specific chemical and biophysical proprieties (e.g., stiffness, nano-, micro- and mesoscale topologies), can interact directly with the biological system affecting cell fate. Not only paracrine signals but also biophysical/mechanical forces experienced by the cells should be taken into consideration during the design of ideal heart valve constructs. 

Natural polymers, such as collagen, fibrin, hyaluronic acid, show important properties since are constituted by macromolecules involved in tissue homeostasis and repair, minimizing, in this way, chronic inflammation and immunological reactions [98]. However, for clinical applications, lot-to-lot variation and low mechanical properties are two strong limitations. To overcome this, synthetic polymers, such as polyglycolic acid (PGA), PLA and PCL, have been widely studied due to their tunable mechanical properties [98]. For example, Kluin et al. developed a bioabsorbable valve based on a supramolecular elastomeric polymer, compatible with both surgical and transcatheter implantation, which maintained its functionality, enhancing cell-driven remodeling (elastic fibers, glycosaminoglycans (GAG) and collagen) up to 12 months [95]. Recent studies have demonstrated that “hybrid materials”, based on natural and synthetic biomaterials, are able to respond efficiently to valve hemodynamic microenvironment and promote homogeneous cell distribution into the scaffold [99]. For example, using a mixture of poly(4-hydroxybutyrate (P4HB) and gelatin /PGA, it was possible to fabricate, through the jet spinning technique, a complete heart valve with a fibrous structure that supported progressive VICs infiltration and tissue growth in vitro [100]. 

**Figure 2:**
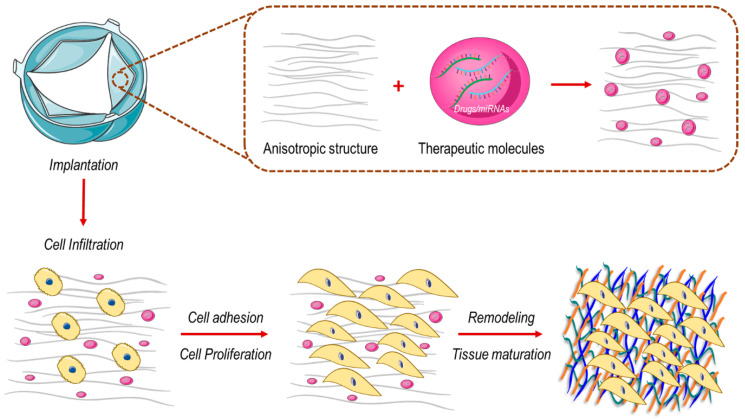
In situ tissue engineered heart valve (TEHV) approach. By the use of this approach, the wound healing cascade—which characterize valve regeneration process—is harnessed for repopulating cell-free synthetic scaffold with living cells. A specific anisotropic structure of the scaffold in combination with specific therapeutic molecules involved in cell mechanosensing (e.g., YAP inhibitors, drugs interfering with stress fibers tensioning) drive towards a scaffold repopulation with proliferative non-activated cells able to synthetize new ECM components (collagen (blue), elastin (green) and GAG (orange)) and thus realize a functional heart valve tissue.

Another solution to overcome the adverse remodeling of cell-free valve implants could be to directly modulate the activity of genes involved in cell mechanosensation and thus reduce the intracellular transmission of mechanical cues, thereby reducing the transition of cells toward a matrix remodeling and compacting phenotypes. Examples may include the employment of materials with finely tuned viscoelastic properties to maintain a relatively low level of intracellular stress fiber tensioning [78,79] or delivering drugs interfering with downstream effectors of the mechanically-activated signal transduction cascades, e.g., the Hippo-dependent transcriptional pathway [101] (Figure 2). Interestingly, it has been found that NOTCH signaling reactivation is required for cardiac valve regeneration in the zebrafish model [102]. Since YAP inhibition induced NOTCH signaling [103], it is possible to speculate again that the inhibition of the mechanosensing machinery in valve interstitial cells might be a viable solution to prevent cell-mediated tissue remodeling, thus promoting valve regeneration.

The in vitro approach to manufacture TEHV consists of the employment of decellularization techniques [104] to produce animal-derived scaffolds naturally containing cell-instructing signals for proper cell differentiation. For example, our laboratory demonstrated the biological compatibility of decellularized human and animal (porcine/bovine) pericardium for in vitro engineering patches of ”living” valve tissue amenable to reconstruct pathologic valves in a prostheses-free manner, also potentially with autologous cells [105,106,107]. Using an alternate perfusion bioreactor, we were able to obtain a homogeneous distribution of proliferative VICs inside the decellularized pericardium, without affecting the structural integrity and the mechanical proprieties of the tissue. Mass spectrometry analysis revealed a de novo synthesis of ECM proteins by the introduced cells compared to the native pericardial membrane. This valve maturation of the tissue constructs was associated also with the growth of cells expressing ”quiescent” VIC phenotypes, expressing low levels of α-smooth muscle actin (α-SMA), a marker of VIC pathological transformation [3], and with YAP prevalently localized in the cytoplasm, probably due to a physiologic compliance of the decelullarized scaffold.

The third approach is represented by the in vivo method, which consists of the implantation of the scaffold directly in the human body, used as a bioreactor. However, this process has possible disadvantages: the collagen-based membrane, formed after the subcutaneous implantation, could be thrombogenic; scar-like tissue formation might cause a lack of valve ECM proteins, such as elastin, affecting tissue architecture and functionality; valve tissue formation and maturation may be long-term [108].

In summary, by integrating various manufacturing technologies with material discoveries and long-term biological characterization, it is possible to realize a customize scaffold able to regulate the cell behavior and to provide the formation of a functional heart valve tissue durable under biomechanical loading.

## 4. Blood Vessels

### 4.1. Flow-Dependent Cellular Mechanotransduction Regulates Pro-Pathological Signaling Pathways in Blood Vessels

As we have already discussed in another contribution (e.g., [17]), mechanical forces generated by blood flow are among the major determinants of vascular morphogenesis and physiology. Both in embryonic and in adult life, alterations in the blood flow induce vessel remodeling through the activation of mechanical-dependent signaling cascades involved in the vessel wall development and homeostasis. In addition to these functions, perturbed hemodynamic conditions can also contribute to a pathologic evolution of vascular tissue, consequent to metabolic, genetic or inflammatory-related injuries.

Under physiological conditions, laminar shear stress (LSS), a tangential force generated by the flowing blood, acts on the endothelial cells surface by promoting vaso-protective effects, through the release of small molecules and cytokines with antithrombotic and vasodilator effects (e.g., nitric oxide (NO)) [109,110]. Indeed, in vitro simulation of shear stress on endothelial cells (ECs) activates multiple mechanosensors located at the cell membrane that modulate the expression and the activity of enzymes, such as the endothelial nitric oxide synthase (eNOS), inducing the release of NO [111]. For example, one of these flow-dependent genes is Krϋppel-like factor (KLF2), which was found to be up regulated in high shear stress regions controlling vascular tone [112]. The flow patterns and hemodynamic forces are not uniform in the vascular system. In straight parts of the arterial tree, blood flow is laminar and wall shear stress is high; in branches and curvatures, flow is disturbed with an irregular distribution of low wall stress [113]. While LSS upregulates the expression of EC genes and proteins with atheroprotective functions [114], disturbed flow with low shear stress determine endothelial dysfunction contributing to the development of vascular pathologies in concert with genetic, biochemical and lifestyle risk factors [115]. Supporting this evidence, the earliest atherosclerotic lesions develop preferentially at arterial branches and curvatures, where the blood flow is disturbed [116]. These conditions promote vascular inflammation through increased endothelial permeability and an enhanced expression of endothelial leukocyte adhesion molecules, favoring the homing of leukocytes [117]. Variations in shear stress are sensed by the endothelium via a mechanosensitive complex on EC membranes, consisting of caveolae, primary cilia and receptors [118]. This machinery converts the mechanical signals in biochemical and transcriptional activation promoting the expression of pro-atherogenic/pro-inflammatory genes [119]. For example, it has been demonstrated in vivo that laminar shear stress-controlled vessel maintenance inducing an actin cytoskeletal reorganization in ECs associated with YAP-TEAD transcriptional activation [120]. 

Disturbed blood flow also affects the stability of the plaque. In this respect, it has been demonstrated that forming plaques create, inside vessels, sections with perturbed flow, which contribute significantly to their rupture [121].

Vascular cells are subjected not only to shear stress but also to hydrostatic pressure and cyclic strain due to pressure patterns, which involve the deeper layers of the vessel wall, such as the media and the adventitia. In particular, the position and the arrangement of vascular smooth muscle cells (VSMCs) inside the vessel wall affect their ability to sense and respond to this oscillatory pulsatile pressure [122]. An interesting example in which altered wall strain forces due to coronary flow pattern contribute to the setting of vascular pathology is the coronary venous bypass grafting [123]. This surgical procedure involves the use of venous conduits, preferentially saphenous veins, which are implanted in peripheral/coronary position in patients with chronic ischemic heart disease. Vein conduits are subjected to a shift from a low and constant flow to a high and pulsatile coronary pattern, which affects both the endothelium, which experiences high and perturbed shear stress, and the VSMCs, due to the high cyclic strain. Direct exposure of human saphenous veins to pulsatile flow determines remodeling in the vessel wall resulting in a conversion of VSMCs from a contractile to a synthetic/proliferative phenotype with a consistent release of Thrombospondin-1 (TSP-1), a matricellular protein involved in the TGF-β pro-fibrotic pathway [123]. This protein has a direct chemotactic effect on saphenous vein adventitial progenitor cells, which migrate from adventitia to the media layer and differentiate in myofibroblast-like cells, thus contributing to the pro-pathologic programming of the graft. This suggests an emerging role for cell-based mechanosensitivity in the vein graft disease, not only related to shear stress-dependent flow but also to a mechano-paracrine mechanism involving the media and the adventitia layers [119].

Taken together, this evidence suggests the importance of a better understanding of the mechanical characteristics of the vessel in order to realize biomaterials suitable for vascular tissue engineering, which take in consideration shear stress, cyclic strain and matrix stiffness.

### 4.2. Mechanical and Structural Characteristics of Tissue Engineered Biomimetic Graft for Vascular Disease Treatment

Despite several technological advances in bioengineering in recent years, there is still intense research to find suitable methods and materials to manufacture a definitive tissue engineered vascular graft (TEVG) with the ability to replicate the functionality of natural vessels [124,125]. The clinical need in this area is extremely high, considering the necessity of more advanced substitutes of autologous vessels (e.g., internal mammary artery or saphenous vein) as coronary or peripheral bypass implants [126]. Although various types of vascular-derived cells—ECs, VSMCs and cells with progenitor characteristics (e.g., pericytes)—may be employed to this aim, the selection of the scaffold composition and geometry is still a major discriminant to provide the suitable microenvironment to promote tissue growth and self-renewal. To date, various materials (synthetic, natural and biosynthetic) and manufacturing processes have been proposed by researchers to meet the structural and mechanical requirements of TEVGs. On the other hand, these options have often shown evident limitations and shortcomings [125], such as mismatches between mechanical properties of the grafts compared to the surrounding vessels and an insufficiency in cellular colonization and self-renewal [127] that lead to graft failure.

Mechanical sensitivity of the cells employed to engineer TEVGs might be exploited to obtain better performances of engineered vascular grafts. For example, a line-oriented scaffold topography along the flow direction is beneficial to achieve cellular orientation along the direction of the flow and, therefore, enhance the formation of a confluent monolayer of endothelial cells at the lumen of the grafts [128,129]. In a second example, Yi et al. also demonstrated that higher stiffness of the scaffold significantly enhanced cell adhesion, even if it increases the risk of pathological differentiation of the ECs, e.g., by endothelial–mesenchymal transition [130]. By contrast, softer fibers and substrates promoted differentiation of vascular progenitor cells into ECs [131], thus showing the importance of the basal lamina compliance to direct correct ECs differentiation in the graft.

Concerning materials, one of the most investigated for manufacturing vascular structures is silk protein component fibroin [132,133]. Compared to other biodegradable materials, silk is a naturally anti-thrombogenic protein [134] that does not elicit severe inflammatory responses [135]. In addition, it can be blended with several other biocompatible materials (e.g., poly-Caprolactone and poly-Lactic-Acid) to obtain hybrid materials with tunable mechanical properties and increased biocompatibility [136]. Silk proteins can be manufactured with different technologies. Examples include electrospinning, a method enabling deposition of polymeric fibers over surfaces or around rotating mandrels that allows creating vascular-like tubular constructs [137] with variable porosity, directly amenable for in vitro cellularization and/or in vivo transplantation, or 3D printing of complex bioinspired constructs [138].

**Figure 3:**
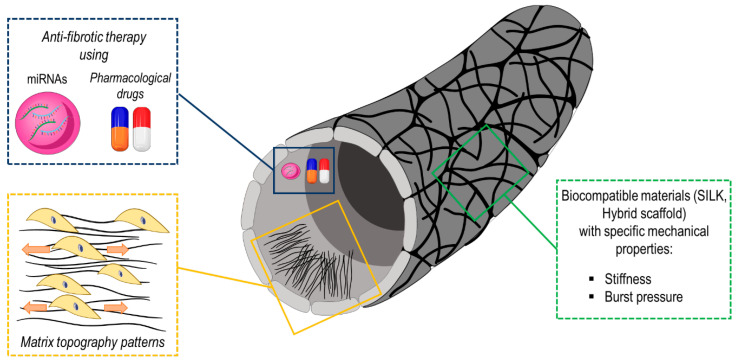
Mechanical properties (e.g., burst pressure, stiffness), surface modifications (e.g., matrix topography patterns, functionalization with specific anti-fibrotic miRNAs) and the selection of the proper biomaterial (e.g., hybrid scaffold, silk) play a crucial role in promoting maturation of vascular tissue engineered constructs.

As in the case of TEHVs, TEVGs can also be manufactured with various strategies depending on the desired application. In a first approach, cells are allowed to grow inside the tubular scaffolds to achieve a full level of cellularization before transplantation [139]. In such a case, a bioreactor is often used to achieve mechanical maturation of the vessel constructs by applying specific flow conditioning regimes able to tailor the blood vessel adaptation to the required (e.g., arterial vs. venous) hemodynamic performance. A second approach consists of transplanting cell-free tubular scaffolds in vivo, exploiting the ability of the recipient body to colonize the grafts with own cells and direct in situ cellularization (Figure 3) [133]. 

Differently from TEHVs, where in situ cellularization with recipient cells able to form the multi-layered structure of the leaflets is still relatively inconsistent, the process of TEVG cellular colonization in vivo leads to a more complete and stable vascular maturation of the constructs. This depends on reparative inflammatory pathways that are activated in the hosts following interposition of the graft in native vessels [140] and by the different nature of the mechanical forces involved in vascular grafts vs. valve implants and the specific effectors involved in their sensing. 

Summarizing, a functional vascular graft material should have: proper mechanical characteristics, in order to withstand pressure and flow forces; suitable suturability and ease of use; a broad availability in different sizes, a good porosity, reasonable manufacturing cost and long term patency [141]. Additionally, bioartificial vascular grafts could be also functionalized with drugs/miRNAs in order to prevent the mechano-dependent development of cardiovascular pathologies. Several miRNAs are known to regulate different vascular cell functions such as cell differentiation, contraction, migration, proliferation (Table 3). In this regard, Ruirui and colleagues found that high levels of miR-21 in neointima hyperplasia promoted VSMC proliferation and decreased VSMC apoptosis [142]. The same research group identified miR-222 as a critical pro-proliferative factor in cultured human VSMCs since it targets p27 (Kip1) and p57 (Kip2) genes compromising their cell cycle inhibitory activity [143]. In the context of atherosclerosis, shear stress regulates endothelial cell activation through miRNAs [144]. For instance, levels of miR-92a are downregulated by laminar shear flow with the consequent increase in its targets, such as KLF2 [145] or KLF4 [146], which are implicated in protection against atherogenesis. Recently, miR-22 has recently been identified as a factor able to influence VSMC phenotypic changes and neointima formation. Evidence suggests that TGF-β is able to transcriptionally modulate miR-22 expression level through a p53-dependant mechanism [147]. Since the TGF-β pathway was found to be upregulated in coronary artery-like mechanical stimulated human veins [123], vascular grafts functionalized with this miRNA could represent an innovative strategy to prevent cell pathological activation leading to altered ECM remodeling and thus vascular fibrosis.

In conclusion, the ideal graft will enforce a biomimetic topography and tunable stiffness providing the proper signaling cues for the cells in order to promote a correct tissue remodeling and to prevent vascular occlusion in consequence of wall strain/stress due to hemodynamic forces.

## 5. Conclusions and Future Perspectives

The present article describes findings supporting the integration of basic knowledge on cell mechanosensation into criteria of bioartificial tissue manufacturing, with the aim at evolving more effective cardiovascular tissue generation/regeneration approaches. This emerging evidence indeed suggests that homeostatic control of cardiovascular tissues depends not only on paracrine pathways acting on cellular renewal vs. pathological programming but also on correct distribution and sensing of mechanical forces by the cells. Alongside the direct consequences of cell motion directly impacting cellular physiology, the passive biophysical characteristics of the tissues, (e.g., mechanical compliance) and the three-dimensional distribution of cell adhesion and cell-to-cell communications establish crucial mechanical-dependent ”checkpoints” ensuring proper cellular integration and self-renewal of bioartificially designed tissues. Finally, the possibility to integrate specific drugs and factors affecting the pathways involved cell mechanoperception in the 3D scaffold design provides a realistic hope to achieve proper arrangement of instructive signals regulating cellular differentiation and reprogramming in a spatially controlled manner. In our view, this approach will contribute to increasing the performance of cardiovascular advanced therapies and will open new avenues for the delivery of durable replacement tissues, overcoming the existing limitations.

## Figures and Tables

**Table 1:** miRNAs involved in the mechano-regulation of cardiac fibrosis.

miRNA	Target Gene	Activity	References
**miR-133**	*CTGF*	Anti-fibrotic	[49]
**miR-30**	*CTGF*	Anti-fibrotic	[49]
**miR-29**	*COL1A1, 1A2,* *FBN, ELN1, PDGFR, TAB1, ADAM*	Anti-fibrotic	[48]
**miR-21**	*PTEN, SMAD7, STAT3*	Pro-fibrotic	[50]
**miR-34**	*SMAD4*	Pro-fibrotic	[51]

**Table 2:** List of miRNAs implicated in mechano-dependent onset of calcific aortic valve disease (CAVD).

miRNA	Target Gene	Activity	References
**miR-30b**	*RUNX2, SMAD1, CASP-3*	Pro-calcification	[58]
**miR-141**	*SMAD2, GATA3*	Anti-calcification	[77]
**miR-34a**	*NOTCH*	Pro-calcification	[81]
**miR-638**	*SP7*	Anti-calcification	[82]

**Table 3:** Circulating miRNAs involved in the pathological programming of different vascular diseases.

miRNA	Target Gene	Functions	References
**miR-21**	*PTEN, BCL-2*	Promotes VSMCs proliferation	[142]
**miR-222**	*Kip-1, Kip-2*	Promotes VSMCs proliferation	[143]
**miR-92a**	*KLF2, KLF4*	Promotes atherogenesis	[145,146]
**miR-22**	*MECP2*	Regulates VSMCs phenotypic modulation	[147]
